# Brain transport platform for precision drug delivery of large molecule therapeutics to treat Alzheimer’s disease

**DOI:** 10.1002/alz70859_102074

**Published:** 2025-12-25

**Authors:** Liqun Wang, Joao Santos, Amy England, Yifei Lu, Amanda Graveline, Melinda Sanchez, Teresa Barata, Daniela Texeira, Donald Ingber, James Gorman

**Affiliations:** ^1^ Harvard University, Boston, MA USA; ^2^ FairJourney Biologics, Porto, Porto Portugal; ^3^ Wyss Institute at Harvard, Boston, MA USA; ^4^ Wyss Institute at Harvard, boston, MA USA; ^5^ Flow EighteenThirtyEight, Porto, Porto Portugal; ^6^ Harvard University, Cambridge, MA USA

## Abstract

**Background:**

Drug delivery to the brain, including to specific diseased brain cells and regions, remains a formidable challenge for the development of effective brain disease treatments. The blood‐brain barrier prevents most therapeutics from reaching the brain. We are developing a diverse panel of brain shuttles to enhance brain uptake and allow targeting to specific cell types and regions within the brain.

**Methods:**

We have developed several panels of brain shuttles, each binding a receptor that is highly abundant on brain microvascular endothelial cells. To rank their transcytosis efficiency, the shuttles were fused to trastuzumab or another IgG with no mouse target. We performed brain uptake assays of each shuttle‐drug fusion in humanized transcytosis receptor knock‐in mice. In each mouse line, the extracellular domain of the mouse TfR, CD98hc, or Target 3 receptor was replaced with its human counterpart. Fusion drug concentrations were assayed in parenchymal brain lysates at time points up to 4 weeks, and their cellular distribution was visualized by immunostaining in brain tissues. Additional shuttle characterizations included human and cynomolgus monkey affinities, epitope binning, sequence liabilities and hematology analysis.

**Results:**

The lead anti‐TfR and anti‐CD98hc brain shuttles increased parenchymal brain uptake of IgGs by 10‐fold or more compared to IgG alone, and showed differentiated brain pharmacokinetics and distribution. The anti‐hTfR brain shuttles crossed the BBB and reached brain parenchyma in 1 day, while the anti‐hCD98hc shuttles showed a slower and more sustained brain exposure for up to 4 weeks, resulting in higher AUC. Anti‐hTfR‐IgG fusion molecules show a prominent neuronal staining pattern, while anti‐hCD98hc‐IgG fusions show diffuse staining in the interstitial space. Target 3 shuttles show slow and sustained brain uptake and prominent neuronal staining. In addition, we evaluated the brain uptake of two published anti‐IGF1R antibodies. We confirmed binding of anti‐IGF‐1R‐trastuzumab fusion molecules to proteins and cells, but we did not observe significantly increased brain uptake in normal mice.

**Conclusion:**

We have developed several panels of efficient antibody brain shuttles. The lead anti‐hTfR, anti‐hCD98hc and anti‐Target 3 brain shuttles can be tailored to improve the brain uptake and target engagement of diverse payloads.

